# Genome Sequences of *Bacillus thuringiensis* Serovar kurstaki Strain BP865 and *B. thuringiensis* Serovar aizawai Strain HD-133

**DOI:** 10.1128/genomeA.01544-16

**Published:** 2017-02-02

**Authors:** Haeyoung Jeong, Soo-Keun Choi, Seung-Hwan Park

**Affiliations:** Infectious Disease Research Center, Korea Research Institute of Bioscience and Biotechnology (KRIBB), and Biosystems and Bioengineering Program, University of Science and Technology (UST), Daejeon, Republic of Korea

## Abstract

We report the draft genome sequences of two insecticidal strains against lepidopteran pests, *Bacillus thuringiensis* serovar kurstaki strain BP865, an isolate from the South Korean phylloplane, and strain HD-133, a reference strain of *B. thuringiensis* serovar aizawai.

## GENOME ANNOUNCEMENT

*Bacillus thuringiensis* is a ubiquitous soil bacterium that produces insecticidal crystal proteins, also known as δ-endotoxins (Cry and Cyt toxins), during the sporulation stage (1). Because each type of *B. thuringiensis* toxin is highly active against specific insect larvae, with little or no effect on humans, such toxins have been widely used as environmentally friendly biopesticides for various crops (2).

Strain BP865 (=KCTC 8689P), an isolate from the phylloplane in South Korea, was shown to exhibit both insecticidal and fungicidal activities (J. I. Kim et al., Korean Intellectual Property Office, 28 April 1997). The biochemical and growth characteristics indicate that BP865 is indistinguishable from HD-1, which is the most widely used bioinsecticide and is recognized as the reference standard in the United States (3). Strains belonging to serovar aizawai, including HD-133, have elicited special interest owing to their production of a “novel” toxin (CryIC family) that is active against *Spodoptera* spp. (4–7).

Genomic DNA preparation was carried out using a Wizard genomic DNA purification kit (Promega, USA) according to the manufacturer’s instructions. Library construction (average insert size of ~400 bp) and genome sequencing were carried out using an Illumina HiSeq 2000 platform at the National Instrumentation Center for Environmental Management (Seoul, Republic of Korea). Sequence totals of 1.59 Gb and 1.62 Gb (2 × 101 cycles) were produced from BP865 and HD-133, respectively. A *de novo* genome assembly was applied using the A5-miseq pipeline (8), version 20160825, which yielded 6,408,259 bp in 225 scaffolds with an *N*_50_ of 99,607 bp for BP865, and 6,438,280 bp in 214 scaffolds with an *N*_50_ of 67,785 bp for HD-133. Genome annotation was carried out using Prokka (9) and NCBI’s Prokaryotic Genome Annotation Pipeline.

Average nucleotide identity analysis using all 78 available *B. thuringiensis* genomes from RefSeq (as of 27 October 2016) indicated that BP865 was closest to serovar kurstaki HD-1 (JMHW01; 99.9%), whereas HD-133 was closest to serovar galleriae HD-29 (CP010089-99; 99.8%), followed by the two serovar aizawai strains Leapio01 (AMXS02; 99.7%) and Hu4-2 (AMXT02; 99.7%). Scaffolds with ≥95% BLAST+ query coverage against complete plasmid sequences for *B. thuringiensis* available from the RefSeq plasmids were placed to putative plasmids. Through this approach, 87 scaffolds from BP865 (910 kb) and 77 scaffolds from HD-133 (687 kb) could be tentatively assigned to the plasmids (http://genoglobe.kr/kribb/two_Bt_strains_2016).

BP865 encodes δ-endotoxins genes for Cry1Aa, Cry1Ab, Cry1Ac, Cry1Ia, and Cry2Ab, whereas HD-133 encodes for Cry1Aa, Cry1Ab, Cry1Ca, Cry1Da, Cry1Ia, Cry2Ab, and Cry9Ea, thereby suggesting that the latter has a wider insecticidal spectrum. However, these include partial or apparently fragmented genes, which necessitates further validation of the complete gene structure. We also identified at least two nonribosomal peptide synthetase (NRPS) genes and two NRPS/polyketide synthetase hybrid genes from both genome assemblies, implying that the potential use of *B. thuringiensis* strains can be extended beyond the biological control of insect pests.

### Accession number(s).

These whole-genome shotgun projects have been deposited in DDBJ/ENA/GenBank under the accession numbers MOXR00000000 (BP865) and MOXQ00000000 (HD-133). The versions described in this paper are the first versions, MOXR01000000 and MOXQ01000000.
